# Development and Validation of a Model for Opioid Prescribing Following Gynecological Surgery

**DOI:** 10.1001/jamanetworkopen.2022.22973

**Published:** 2022-07-20

**Authors:** Isabel V. Rodriguez, Paige McKeithan Cisa, Karen Monuszko, Julia Salinaro, Ashraf S. Habib, J. Eric Jelovsek, Laura J. Havrilesky, Brittany Davidson

**Affiliations:** 1 Department of Obstetrics and Gynecology, Duke University, Durham, North Carolina; 2 Department of Obstetrics and Gynecology, University of Washington, Seattle; 3 Department of Anesthesiology, Duke University, Durham, North Carolina

## Abstract

**Importance:**

Overprescription of opioid medications following surgery is well documented. Current prescribing models have been proposed in narrow patient populations, which limits their generalizability.

**Objective:**

To develop and validate a model for predicting outpatient opioid use following a range of gynecological surgical procedures.

**Design, Setting, and Participants:**

In this prognostic study, statistical models were explored using data from a training cohort of participants undergoing gynecological surgery for benign and malignant indications enrolled prospectively at a single institution’s academic gynecologic oncology practice from February 2018 to March 2019 (cohort 1) and considering 39 candidate predictors of opioid use. Final models were internally validated using a separate testing cohort enrolled from May 2019 to February 2020 (cohort 2). The best final model was updated by combining cohorts, and an online calculator was created. Data analysis was performed from March to May 2020.

**Exposures:**

Participants completed a preoperative survey and weekly postoperative assessments (up to 6 weeks) following gynecological surgery. Pain management was at the discretion of clinical practitioners.

**Main Outcomes and Measures:**

The response variable used in model development was number of pills used postoperatively, and the primary outcome was model performance using ordinal concordance and Brier score.

**Results:**

Data from 382 female adult participants (mean age, 56 years; range, 18-87 years) undergoing gynecological surgery (minimally invasive procedures, 158 patients [73%] in cohort 1 and 118 patients [71%] in cohort 2; open surgical procedures, 58 patients [27%] in cohort 1 and 48 patients [29%] in cohort 2) were included in model development. One hundred forty-seven patients (38%) used 0 pills after hospital discharge, and the mean (SD) number of pills used was 7 (10) (median [IQR], 3 [0-10] pills). The model used 7 predictors: age, educational attainment, smoking history, anticipated pain medication use, anxiety regarding surgery, operative time, and preoperative pregabalin administration. The ordinal concordance was 0.65 (95% CI, 0.62-0.68) for predicting 5 or more pills (Brier score, 0.22), 0.65 (95% CI, 0.62-0.68) for predicting 10 or more pills (Brier score, 0.18), and 0.65 (95% CI, 0.62-0.68) for predicting 15 or more pills (Brier score, 0.14).

**Conclusions and Relevance:**

This model provides individualized estimates of outpatient opioid use following a range of gynecological surgical procedures for benign and malignant indications with all model inputs available at the time of procedure closing. Implementation of this model into the clinical setting is currently ongoing, with plans for additional validation in other surgical populations.

## Key Points

**Question:**

Can patient-specific factors be incorporated into an inclusive model to predict postoperative opioid use?

**Findings:**

In this prognostic study of patients from a single academic gynecologic oncology practice, a model was developed and internally validated to predict number of opioid pills used following hospital discharge using the following components: age, educational attainment, smoking history, anticipated pain medication use, anxiety regarding surgery, operative time, and preoperative pregabalin administration.

**Meaning:**

These findings suggest that a model including patient-elicited and surgery-specific factors can predict outpatient opioid use following a range of gynecological surgical procedures.

## Teaser

This prognostic study develops and validates a model for predicting outpatient opioid use after a range of gynecological surgical procedures.

## Introduction

The misuse and diversion of prescription opioids has contributed to the current epidemic of the opioid use disorder in the US.^1^ Provisional data indicate that this health crisis has worsened in the past 2 years, with more than 77 000 opioid-related deaths reported in the 12-month period ending in December 2021.^2,3^ Most cases of chronic opioid use disorder start with a legitimately obtained prescription.^4^ Prescription opioids are one of the mainstays of pain management following surgery; however, studies have shown that surgical specialists routinely overprescribe opioid medications postoperatively.^5,6,7^ Approximately 6% to 8% of opioid-naive patients develop chronic use following surgery,^8^ and the probability increases with duration of opioid therapy.^9,10^ It is difficult to balance adequate postoperative pain control with limiting opioid prescription dosing,^11^ because poorly controlled pain ultimately prolongs recovery^12^ and is a significant predictor of chronic pain.^13^ These factors lead to indiscriminate pain management plans to control pain for most patients.

More-precise prescribing tools may aid clinicians in navigating this challenge. The literature demonstrates efforts for individualized postoperative opioid prescribing practices,^14,15,16,17,18^ although not all of these tools have been appropriately validated,^19^ and they have limited generalizability. Although these studies demonstrate potential alternatives to the one-size-fits-all practice of opioid prescribing, an inclusive model applicable to a broad patient population is needed. In the current prognostic study, we sought to develop and validate a model predicting opioid use after hospital discharge for patients undergoing a range of gynecological procedures.

## Methods

### Recruitment

We prospectively enrolled 2 cohorts of patients undergoing surgery within the gynecologic oncology division of an academic institution: a training cohort from February 1, 2018, to March 1, 2019 (cohort 1), and a testing cohort for internal model validation from May 2019 to February 2020 (cohort 2). Participants were eligible if they were aged 18 years or older, English speaking, and scheduled for open, laparoscopic, or robotic-assisted abdominal surgery. Participants were excluded if they were undergoing only vaginal or vulvar surgery. Baseline opioid use, defined as self-reported daily use before surgery, was not exclusionary. The sample size required to estimate the entire distribution of the response with a global margin of error not exceeding 0.1 was 184 participants; therefore, we planned to enroll approximately 250 per cohort to account for loss of follow-up and rescheduled or canceled surgical procedures.

Written informed consent was obtained from all participants at the time of enrollment. This study was approved by Duke University’s institutional review board and follows the Transparent Reporting of a Multivariable Prediction Model for Individual Prognosis or Diagnosis (TRIPOD) reporting guideline.^19^

### Study Activities

Participants completed a preoperative survey including baseline characteristics, the Pain Catastrophizing Scale,^20^ and questions regarding preoperative opioid use. Participants were also asked about preoperative anxiety and expectations for postoperative pain.^16^ Participants self-identified race and ethnicity, and we collected this for the purpose of characterizing the study population. Clinical practitioners assigned Eastern Cooperative Oncology Group performance status.^21^ Routine intraoperative care was administered at the discretion of the individual’s anesthesia and surgical teams. All patients were managed via our institution’s enhanced recovery after surgery (ERAS) protocol, which included standardized preoperative acetaminophen, gabapentinoid depending on patient age and liver and kidney function, and placement of a thoracic epidural if laparotomy was anticipated. Postoperative pain management included scheduled acetaminophen and ibuprofen as appropriate, as well as oral and intravenous opioid pain medication according to operative approach. The type and amount of all outpatient prescriptions were at the discretion of the discharging practitioner. Patients received instructions advising scheduled use of over-the-counter pain medications with opioid medications as needed, as well as a pill diary as a memory aid to reference during follow-up surveys.

After hospital discharge, research staff contacted participants weekly to complete surveys until the participant reported no longer using opioid medications or 6 weeks after surgery, whichever came first. For baseline opioid users, the surveillance period ended when they returned to preoperative opioid use. We chose the 6-week limit for the surveillance period because patients in our practice are routinely scheduled for follow-up visits between 3 and 6 weeks after surgery and assessment could be performed for postoperative complications. Follow-up was considered complete once the number of remaining opioid pills from all postoperative prescriptions was documented. Postoperative surveys assessed pain scores, use of opioid and adjunct pain medication, and plans for opioid disposal. Participants or the research team abstracted the data directly into the REDCap electronic data capture tool.^22^ Data abstracted from the electronic health record are outlined in eAppendix 1 in the Supplement.

### Statistical Analysis

The response variable for model development was number of opioid pills used postoperatively, and the primary outcome was model performance as defined by ordinal concordance (C) statistic and Brier score. Secondary outcomes included number of opioid pills prescribed, adequacy of postoperative pain regimen (defined as need for prescription refills, patient satisfaction, and readmissions for pain control), and plan for unused opioids.

We considered all data available before hospital discharge as possible predictor variables (eAppendix 2 in the Supplement). Imputation of missing predictor values was performed using multivariate imputation by chained equation.^23^ We performed exploratory analysis using scatterplot matrices and histograms of univariate and bivariate associations. Because the mean and variance of our response variable was overdispersed, we fit negative binomial, 0-inflated negative binomial, and ordinal regression models to the data. We used an initial unsupervised variable selection approach consisting of unsupervised redundancy analysis and hierarchical clustering. Final predictors were further selected using backward elimination of 1000 bootstrapped samples or using penalized ordinal regression with regularization using 10-fold cross-validation repeated 3 times from the R package ordinalNet.^24^ During model fitting, linear assumptions of continuous variables were relaxed using restricted splines with 3 and 5 knots. For ordinal regression, we verified assumptions of proportionality using visual plots and the Brant test.^25^

We calculated overall model performance using the C statistic. One thousand bootstrapped samples were also used to internally validate model performance (discrimination and calibration) by estimating overfitting. We calculated bias-corrected C statistics for discriminating between pairs of several clinically useful dichotomous outcome thresholds (eg, <5 pills vs ≥5 pills, <10 pills vs ≥10 pills, and <15 pills vs ≥15 pills), and bias-corrected calibration plots were used to visualize the accuracy of the predictions compared with actual pills taken across the entire range of possible predictions. Overall performance at each threshold was assessed using the Brier score. Analyses were performed using R statistical software version 3.5.2 (R Project for Statistical Computing).^26^

Predictions were generated using 3 models: a model produced using backward elimination using 3 predictors, a penalized ordinal regression model using 5 predictors, and a model combining the predictors from the first 2 models using a total of 7 predictors. Data from the internal validation cohort were separately used to generate predictions using these 3 models with the dichotomous outcome thresholds described already.

For final clinical use, we took advantage of all data from both study cohorts and updated each model using a combined data set including the training and testing cohorts. This model was placed into an online calculator for clinical use. Data analysis was performed from March to May 2020.

## Results

**Figure 1.**
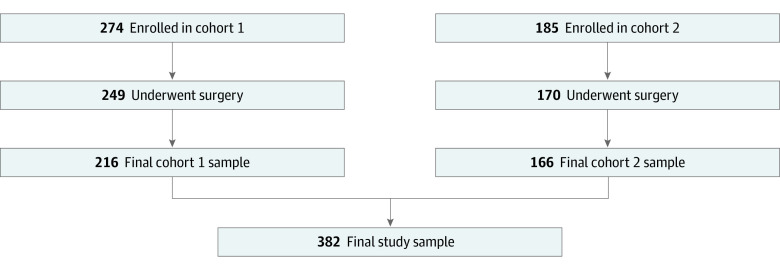
Cohort Enrollment Flowchart Participants were considered ineligible if surgery was canceled, did not occur within the study period, or if the participant underwent nonapplicable surgery. Follow-up was incomplete if total number of opioid tablets used following hospital discharge was not reported.

A total of 459 female adult participants were enrolled in the study; 42 were ineligible because of canceled or nonapplicable surgery or withdrew (Figure 1), leaving 382 patients (91%) (mean age, 56 years; range, 18-87 years) who completed the study and were included in the analysis (216 participants in cohort 1 with a mean [SD] age, of 54 [13] years and 166 participants in cohort 2 with a mean [SD] age of 58 [14] years). Thirty-five participants (7.6%) did not complete the study (their demographic characteristics are displayed in the eTable in the Supplement). Baseline characteristics of participants in cohorts 1 and 2 who completed the study were similar, including education, body mass index, history of tobacco use, history of mental illness, Eastern Cooperative Oncology Group functional status, and American Society of Anesthesiologists score (Table 1). Six participants in cohort 1 (3%) and 17 participants in cohort 2 (10%) reported baseline opioid use. This population had multiple medical comorbidities; 119 patients (55%) in cohort 1 and 93 patients (56%) in cohort 2 had an American Society of Anesthesiologists physical status of 3, indicating severe systemic disease.^27^

**Table 1.** Baseline Characteristics

Characteristic	Patients, No. (%) (N = 382)	
	Cohort 1 (n = 216)	Cohort 2 (n = 166)
Age, mean (SD) [range], y	54.7 (13) [21-84]	58.6 (14) [19-87]
Race		
American Indian or Alaska Native	4 (2)	2 (1)
Asian	7 (3)	5 (3)
Black	49 (23)	36 (22)
White	154 (71)	122 (74)
Other^a^	2 (1)	1 (1)
Ethnicity, Hispanic or Latinx	6 (2)	1 (1)
Education		
High school or less	56 (26)	38 (23)
Some college	43 (20)	36 (22)
Associate degree	20 (9)	11 (6)
Bachelor’s degree	57 (26)	40 (24)
Master’s degree or higher	39 (18)	41 (25)
Baseline opioid use	6 (3)	17 (10)
Body mass index, mean (SD)^b^	32 (8.4)	31.9 (8.6)
Tobacco use		
Never	134 (62)	105 (63)
Former	56 (26)	46 (28)
Current	26 (12)	15 (9)
History of mental illness		
Anxiety	16 (7)	11 (6)
Depression	33 (15)	14 (8)
Anxiety and depression	13 (6)	12 (7)
Eastern Cooperative Oncology Group functional status		
0	84 (39.0)	80 (48.2)
1	128 (60.0)	85 (51.2)
2	2 (1.0)	1 (0.6)
American Society of Anesthesiology score		
1	9 (4)	3 (2)
2	87 (40)	70 (42)
3	119 (55)	93 (56)

^a^ Refers to participants who self-identified as more than 1 race or declined to answer.

^b^ Body mass index is calculated as weight in kilograms divided by height in meters squared.

Surgical and postoperative medication characteristics are displayed in Table 2. The median (IQR) operative time was 139 (100-177) minutes in cohort 1 and 158 (113-204) minutes in cohort 2. The proportion of surgical procedures via a minimally invasive approach was similar in cohort 1 and cohort 2 (73% [158 patients] vs 71% [118 patients]). The most common indications for surgery were known malignant neoplasm (39% [84 patients] vs 49% [82 patients]) and pelvic mass (35% [75 patients] vs 30% [51 patients]), and the most common primary procedure performed was hysterectomy (70% [151 patients] vs 73% [121 patients]). Most participants received preoperative acetaminophen (84% [182 patients] vs 89% [148 patients]). Preoperative gabapentinoid use was lower in cohort 2 (64% [138 patients] vs 16% [26 patients]), including pregabalin use (22% [48 patients] vs 7% [11 patients]). Intraoperative adjunct pain measures included thoracic epidural analgesia (16% [35 patients] vs 25% [42 patients]) and transversus abdominis plane block (11% [23 [patients] vs 4% [6 patients]).

**Table 2.** Surgical and Postoperative Medication Characteristics

Characteristic	Patients, No. (%) (N = 382)	
	Cohort 1 (n = 216)	Cohort 2 (n = 166)
Operative time, median (IQR), min	139 (100-177)	158 (113-204)
Surgical approach		
Minimally invasive	158 (73)	118 (71.0)
Open	28 (13)	24 (14.5)
Converted to open	30 (14)	24 (14.5)
Primary procedure		
Hysterectomy	151 (70)	121 (73)
Adnexal surgery	56 (26)	37 (22)
Other	9 (4)	8 (5)
Indication for surgery		
Pelvic mass	75 (35)	51 (30)
Cancer prophylaxis	15 (7)	17 (10)
Malignant neoplasm	84 (39)	82 (49)
Fibroid uterus	9 (4)	8 (5)
Abnormal uterine bleeding	8 (4)	20 (12)
Pelvic pain	2 (1)	6 (4)
Other	36 (17)	10 (6)
Enhanced recovery after surgery components		
Preoperative acetaminophen	182 (84)	148 (89)
Preoperative gabapentin	90 (42)	15 (9)
Preoperative pregabalin	48 (22)	11 (7)
Preoperative celecoxib	22 (10)	93 (56)
Thoracic epidural	35 (16)	42 (25)
Transversus abdominus plane block	23 (11)	6 (4)
Length of hospital stay, median (range), d	1 (0-17)	1 (0-8)
Prescribed opioids at discharge	211 (98)	153 (92)
Type of opioid prescribed^a^		
Oxycodone 5 mg	188 (89)	138 (90)
Tramadol 50 mg	16 (8)	10 (7)
Hydrocodone 5 mg	2 (1)	2 (1)
Hydromorphone 2 mg	2 (1)	2 (1)
Prescribed pills, mean (IQR), No.	19 (15-25)	13 (10-15)
Required refill	15 (7)	7 (5)
Tablets used, mean (SD), No.	8.0 (10.6)	6.1 (9.0)
Participants using no opioids postoperatively	78 (36)	69 (41)
Opioid disposal plan		
Keep leftover pills	37 (17)	27 (16)
Dispose at home	25 (12)	14 (8)
Drop off at a Take Back Program	50 (23)	22 (13)
I do not know or other	88 (41)	41 (25)

^a^ Opioids prescribed to more than 2 participants are not listed. There were 3 participants in cohort 1 and 1 in cohort 2 prescribed an opioid not listed in the table.

Nearly all participants (211 patients [98%] in cohort 1 and 153 patients [92%] in cohort 2) were prescribed an opioid medication for pain management at the time of hospital discharge, most commonly oxycodone 5 mg (326 patients [85%] among all participants). The median (IQR) number of pills prescribed at the time of discharge was 19 (15-25) in cohort 1 and 13 (10-15) in cohort 2. The proportion of participants who did not use any opioid medication following discharge was 38% overall (147 patients), and the mean (SD) number of opioid pills used was 7 (10) (median [IQR], 3 [0-10] pills). Patient-reported median (IQR) pain score on a scale from 1 to 10 a week following surgery was 2 (1-4). Twenty-two study participants (6%) required a medication refill. There were no readmissions for pain control. After cessation of postoperative opioid prescriptions, only 111 participants (29%) had a disposal plan for unused opioids. Two participants were still using opioid medication 6 weeks after surgery, neither of whom had a history of baseline opioid use preoperatively. Both participants had known malignant neoplasms before surgery. One participant had a surgery that included multiple bowel resections and ileostomy creation, and the other had a postoperative course complicated by wound dehiscence and bowel obstruction.

### Training Cohort

Three different cumulative probability ordinal models had the best fit. One model used backward elimination and had 3 predictors: age, total operating time, and participant’s preoperative anticipated need for pain medication. The overall C statistic was 0.63 (95% CI, 0.59-0.66), and the Brier score was 0.17. A second model was a penalized ordinal regression model, which included 5 predictors: participant’s anticipated need for pain medication (self-reported), whether pregabalin was administered preoperatively, educational attainment, smoking history, and preoperative anxiety regarding surgery. This model had an overall C statistic of 0.63 (95% CI, 0.59-0.67) and a Brier score of 0.16. Because each model had similar discriminatory ability but different predictors, the predictors were all included in a third combined model. The combined model had an overall C statistic of 0.65 (95% CI, 0.61-0.69) and a Brier score of 0.16.

### Testing Cohort

In the testing cohort, the combined model had the best overall discrimination with a C statistic of 0.66 (95% CI, 0.57-0.74) for predicting 5 or more pills (Brier score, 0.21), 0.68 (95% CI, 0.58-0.77) for predicting 10 or more pills (Brier score, 0.16), and 0.69 (95% CI, 0.57-0.79) for predicting 15 or more pills (Brier score, 0.12). The C statistic at the median value of 2 or more pills was 0.66 (95% CI, 0.58-0.74), and the Brier score was 0.25. This model had the best calibration across the range of predicted probabilities (eFigure in the Supplement). The backward stepdown model performed similarly, with a C statistic of 0.68 (95% CI, 0.60-0.76) for predicting 5 or more pills (Brier score, 0.21), 0.71 (95% CI, 0.61-0.80) for predicting 10 or more pills (Brier score, 0.16), and 0.75 (95% CI, 0.64-0.83) for predicting 15 or more pills (Brier score, 0.11). The C statistic at the median value was 0.70 (95% CI, 0.61-0.77) with a Brier score of 0.23. Calibration and overall performance for the backward stepdown model were not as good across the range of pill thresholds (eFigure in the Supplement). The penalized ordinal regression model was not well calibrated in this cohort and, therefore, was not used.

### Final Model Creation

**Figure 2.**
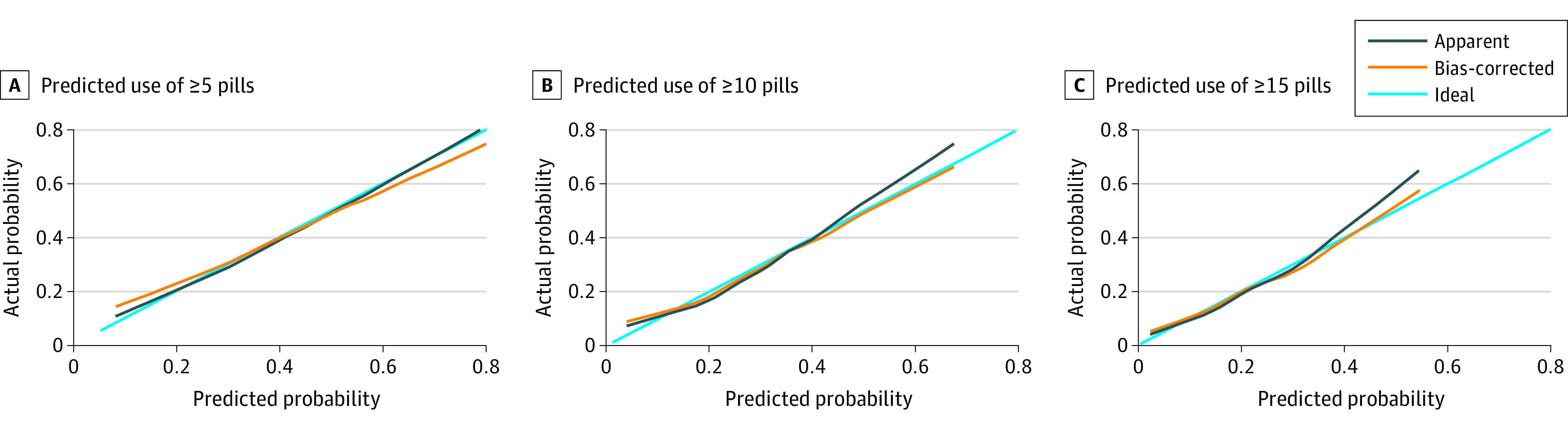
Actual vs Predicted Probability in 3 Models The calibration plot of the combined model is shown when predicting use of 5 or more pills (A), 10 or more pills (B), and 15 or more pills (C).

**Figure 3.**
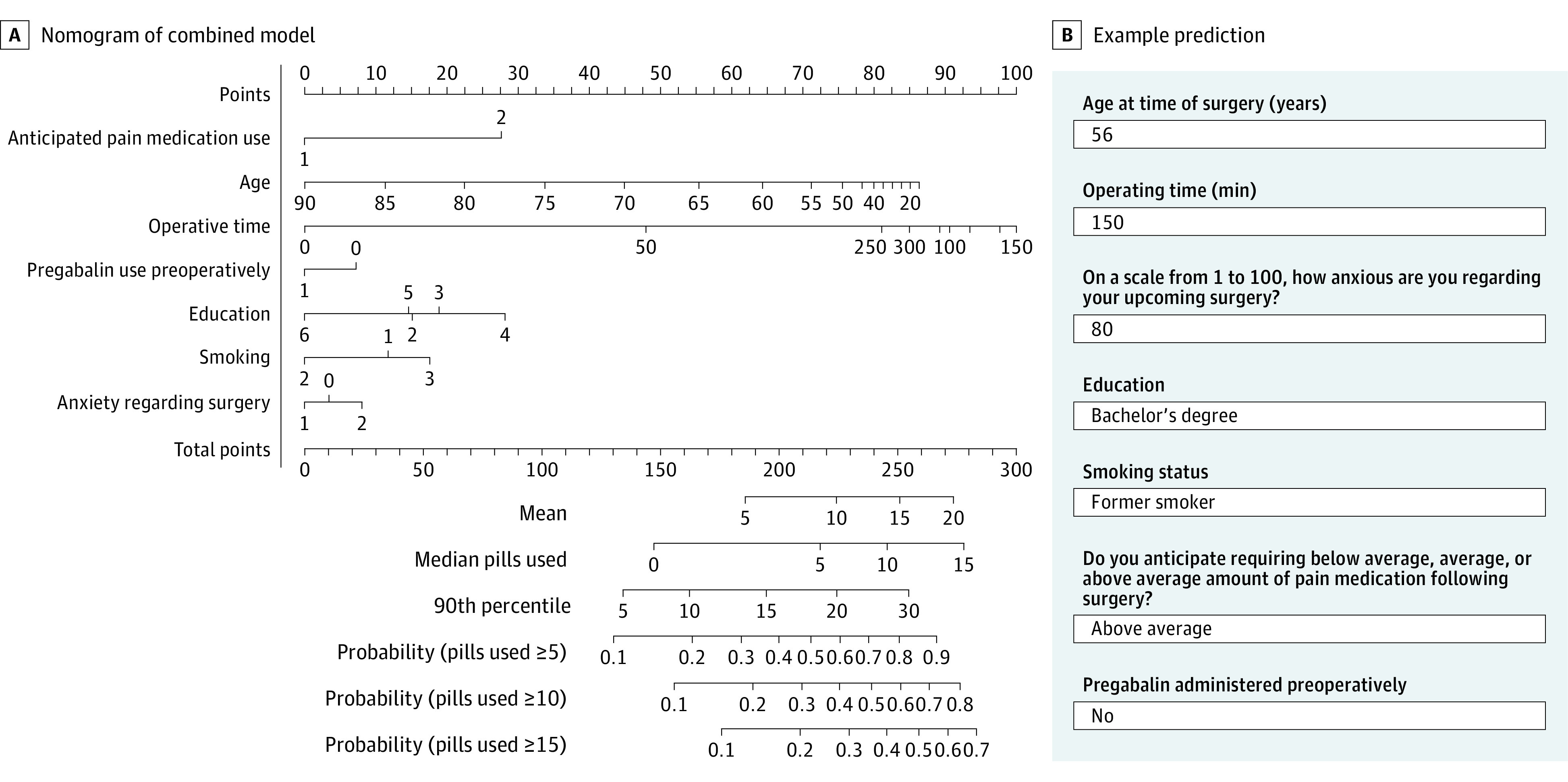
Nomogram of the Combined Model Including the 7 Final Predictors and an Example Prediction A, The nomogram outputs include the mean, median, and 90th percentile for total predicted opioid tablets a patient will use postoperatively, as well as the probability that patients will use 5, 10, or 15 tablets of opioid medication. A key is provided in eAppendix 3 in the Supplement. B, Patients with these characteristics are predicted to use a mean of 12 pills. Although 60% of patients with this history would be predicted to use 5 or more pills, less than 30% would use 15 or more pills.

We then updated the combined and backward stepdown models after adding the first and second patient cohorts (382 patients), and each model was updated to evaluate performance for final use. The combined model was chosen on the basis of a combination of C statistic, calibration, and anticipated model acceptability by clinicians. The performance of the updated combined model improved as expected with the larger sample size. Figure 2 shows the final calibration curves using the combined model. The C statistic was 0.65 (95% CI, 0.62-0.68) for predicting 5 or more pills (Brier score, 0.22), 0.65 (95% CI, 0.62-0.68) for predicting 10 or more pills (Brier score, 0.18), and 0.65 (95% CI, 0.62-0.68) for predicting 15 or more pills (Brier score, 0.14). The C statistic at the median value of 3 more pills was 0.65, with a Brier score of 0.23. A nomogram predicting the median number of opioid pills required for a patient with certain characteristics was created for clinical use using the combined model (Figure 3 and eAppendix 3 in the Supplement), which was adapted to an online calculator.^28^

## Discussion

In this prognostic study, we developed and validated a model for predicting the number of opioid pills taken after gynecological surgery that uses factors easily obtained from health records (age, educational attainment, smoking history, preoperative pregabalin administration, and total operative time), as well as preoperative survey questions (anticipated pain medication use and anxiety regarding surgery). All 7 predictors are available on the day of surgery, making this model feasible to implement following inpatient and outpatient surgery, and this model is agnostic regarding surgical route, intended for use after either open or minimally invasive abdominal surgery. In addition to pill estimation, our model provides probabilities that the patient will use 5 or more, 10 or more, or 15 or more pills to further inform prescribing decisions. This may be helpful when underestimation of use would place additional burdens on the patient, such as practice locations where electronic prescribing is not available.

Our results are consistent with prior studies^5,6,7^ showing that practitioners overprescribe opioids, as 38% of our participants used no opioid medication following surgery. There was a decrease in mean number of pills prescribed to participants in cohort 1 vs cohort 2, which likely reflects increased practitioner education and awareness surrounding opioid prescribing that was concurrent with but not part of our study. The median pain score 1 week after surgery was 2 of 10, suggesting patient satisfaction with their regimen and providing a baseline for comparison in future studies. Only 29% of participants had a disposal plan for unused opioid pills at the end of the surveillance period, and interventions at this stage should also be considered to help reduce opioids in the community.

Our model is similar to the Post-operative Opioid Calculator for Hysterectomy developed by Wong et al,^13^ in that it incorporates components of the patient’s medical history as well as an assessment of expectations for surgery. Preoperative anxiety has been demonstrated in previous studies^16,29^ to be predictive of postoperative pain, and both models highlight the importance of including psychosocial factors. In contrast, our study had a more generalizable population, including patients undergoing laparotomy and patients with suspected or confirmed malignant neoplasm. Given that patients with cancer have been shown to have a higher rate of persistent postoperative opioid use,^8^ this is an important population to study. Additionally, we used TRIPOD reporting guidelines designed to ensure predictive accuracy,^19^ internally validated all models developed in this study, and assessed multiple measures of model performance.

Several investigators have reported rule-based models, such as prescribing according to the number of opioid tablets used before hospital discharge.^15,17,18^ These studies are less applicable to patients discharged the same day, which is an increasingly common practice following minimally invasive surgery and represents the majority of patients undergoing minimally invasive hysterectomy in several studies.^30,31,32^ Guidelines based solely on surgical approach also have limitations. A recent prospective cohort study^33^ suggests that gynecological patients use similar amounts of pain medication following both minimally invasive and open surgical procedures. In our study, surgical approach was considered as a candidate predictor but ultimately was not in the final model. This does not mean that surgical approach is not predictive. Operative time is related to surgical approach, and because of its continuous nature, operative time was more informative, with less prediction error and fewer degrees of freedom. Preoperative pregabalin administration was predictive in our model; however, after 2020 this was removed from our institution’s ERAS protocol and fewer participants in cohort 2 received this medication as part of their standard care compared with cohort 1 (7% vs 22%). Preoperative gabapentinoids have been shown to reduce postoperative pain and opioid consumption^34,35,36^ and remain a part of multimodal analgesia approach at other institutions; therefore, we elected to keep this in our model. We also considered additional components of ERAS protocols, such as use of epidural analgesia, transversus abdominis plane blocks, and others described in the Methods section. These factors did not add predictive power in the final model after controlling for all other predictors; however, one must use caution in concluding that these additional predictors would not have any predictive power when considered in alternate models.

### Limitations

Although we had broad inclusion criteria, all participants were recruited through a gynecologic oncology practice. Gynecologic oncology was chosen to develop and pilot this model because of the high surgical volume and heterogenous patient population; however, further exploration of this model is needed in other patient populations. Despite offering multiple modalities for postoperative follow-up, 35 participants (7.6%) did not complete the study (eTable in the Supplement). Of note, there was a greater percentage of participants from racial and ethnic minority groups in this group. Disparities in pain management affecting racial minority groups are well documented,^37,38,39^ and we as researchers need to consider how a history of inequity may impact desire to participate in research. Our study also relied on self-reporting of opioid use. Because of the societal stigma surrounding addiction and opioid use, some participants may not have honestly reported their opioid medication use preoperatively or postoperatively. Nearly all participants in our study were prescribed oxycodone 5-mg tablets at the time of discharge; therefore, our model is most predictive of use for this medication. Conversion via milligram morphine equivalents would allow for prescription guidance of other opioids.

## Conclusions

The proposed patient-centered model allows for prediction of postoperative opioid use with easily obtained patient-level variables and can be used following a broad range of gynecological procedures. We are currently externally validating this model as a prescribing intervention in an expanded gynecology cohort, with a goal of reducing excess opioids in the community.

## Notes

Supplement. **eAppendix 1.** Electronic Health Record Abstraction **eAppendix 2.** List of 39 Candidate Predictors **eFigure. **Calibration Curves for Testing Cohort **eAppendix 3.** Nomogram Key: Final Predictors **eTable.** Baseline Characteristics of Enrolled Participants Who Did Not Complete the Study
