# Supplementary material for: Development and Validation of a Model for Opioid Prescribing Following Gynecological Surgery
Source: JAMA Netw Open. 2022 Jul 20;5(7):e2222973. doi: 10.1001/jamanetworkopen.2022.22973 (PMC9301519; doi:10.1001/jamanetworkopen.2022.22973)
Supplement: Supplement. — eAppendix 1. Electronic Health Record Abstraction eAppendix 2. List of 39 Candidate Predictors eFigure. Calibration Curves for Testing Cohort eAppendix 3. Nomogram Key: Final Predictors eTable. Baseline Characteristics of Enrolled Participants Who Did Not Complete the Study [file jamanetwopen-e2222973-s001.pdf]

Supplemental Online Content

Rodriguez IV, Cisa PM, Monuszko K, et al. Development and validation of a model for opioid prescribing following gynecological surgery. *JAMA Netw Open*. 2022;5(7):e2222973. doi:10.1001/jamanetworkopen.2022.22973

eAppendix 1. Electronic Health Record Abstraction
eAppendix 2. List of 39 Candidate Predictors
eFigure. Calibration Curves for Testing Cohort
eAppendix 3. Nomogram Key: Final Predictors
eTable. Baseline Characteristics of Enrolled Participants Who Did Not Complete the Study

This supplemental material has been provided by the authors to give readers additional information about their work.

© 2022 Rodriguez IV et al. *JAMA Network Open.*

eAppendix 1. Electronic Health Record Abstraction

Medical history: age, pre-operative diagnosis, history of substance abuse, mental health diagnoses, smoking status, body mass index (BMI)

Surgical/inpatient information: number of intra-abdominal surgeries prior to the scheduled procedure, primary procedure, indication for surgery, whether lymph node dissection and/or bowel surgery was performed, surgical approach, total procedure time, intraoperative/postoperative complications, length of postoperative admission, average pain score during admission, opioid and non-opioid pain medications administered pre-operatively, intra-operatively, or postoperatively during hospitalization

Outpatient postoperative information: opioid medication prescriptions provided at discharge, any additional opioid prescriptions provided after hospital discharge, number of postoperative provider visits and chief complaints at these visits, number of postoperative urgent care or emergency department visits and chief complaints of these visits, and number of hospital readmissions and chief complaint.

© 2022 Rodriguez IV et al. *JAMA Network Open.*

eAppendix 2. List of 39 Candidate Predictors

Race (Asian, Black, White, Other)
Hispanic (yes, no)
Education attainment (High school or less, Some college, Associates degree, Bachelor's degree, Masters or Professional degree)
Number of alcoholic drinks consumed weekly
Consumption of alcoholic drinks weekly (yes, no)
History of chronic narcotic use (yes, no)
Anxiety regarding surgery on a scale of 0 to 100
Anxiety regarding surgery (yes, no)
Anticipated postoperative pain on a scale of 0 to 100
Anticipated need for pain medication (below average, average, above average)
Pain catastrophizing scale: metric describing rumination over pain
Pain catastrophizing scale: metric describing magnification of pain consequences
Pain catastrophizing scale: metric describing feeling overwhelmed by pain
Pain catastrophizing scale: total score
Age (years)
Smoking history (never smoker, former smoker, current smoker)
ECOG score at pre-operative appointment (0, 1, 2, 3)
Number of prior intra-abdominal surgeries
ASA score (1, 2, 3, 4)
Total amount Tylenol administered pre-operatively (amount in mg)
Total amount Gabapentin administered pre-operatively (amount in mg)
Total amount Lyrica administered pre-operatively (amount in mg)
Total amount Celecoxib administered pre-operatively (amount in mg)
ERAS protocol (yes, no)
Mid-thoracic epidural (yes, no)
Intraoperative IV Tylenol administered (amount in mg)
Intraoperative IV Toradol administered (amount in mg)
Intraoperative TAP block (yes, no)
Primary procedure (hysterectomy +/- adnexal surgery, adnexal surgery alone, other)
Lymph node dissection performed (yes, no)
Bowel surgery performed (yes, no)
Surgical approach (laparoscopic, robotic, open, MIS converted to open)
Indication for surgery: pelvic mass
Indication for surgery: cancer prophylaxis
Indication for surgery: malignancy
Indication for surgery: fibroid uterus
Indication for surgery: abnormal uterine bleeding
Indication for surgery: other
Total operative time (mins)

© 2022 Rodriguez IV et al. *JAMA Network Open.*

eFigure. Calibration Curves for Testing Cohort

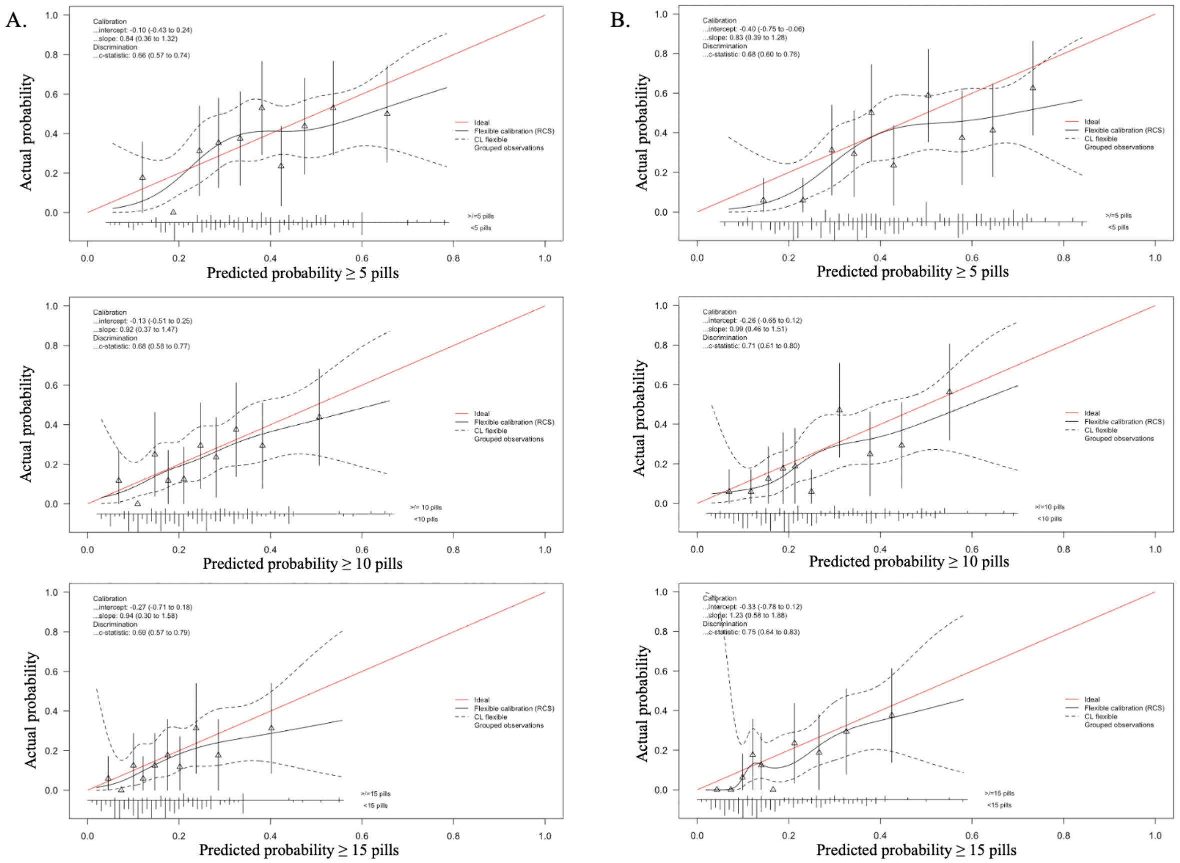

A. Calibration curves for the combined model at the three dichotomous outcome thresholds. B. Calibration curves for the backwards stepdown model.

© 2022 Rodriguez IV et al. *JAMA Network Open.*

eAppendix 3. Nomogram Key: Final Predictors

Anticipated need for pain medication (0 = below average, 1 = average, 2 = above average)
Age (years)
Total operative time (mins)
Pre-operative pregabalin administration (0 = no, 1 = yes)
Education attainment (2 = High school or less, 3 = Some college, 4 = Associates degree, 5 = Bachelor's degree, 6 = Masters or Professional degree)
Smoking history (1 = never smoker, 2 = former smoker, 3 = current smoker)
Anxiety regarding surgery on a scale of 0 to 100 (0 = 0 - 32, 1 = 33 - 66, 2 = 67 - 100).

© 2022 Rodriguez IV et al. *JAMA Network Open.*

eTable. Baseline Characteristics of Enrolled Participants Who Did Not Complete the Study

	Cohort 1 (n=26)		Cohort 2 (n=21)	
Characteristic	N (%)	*p*[b]	N (%)	*p*[b]
Race		0.043*		0.001*
American Indian or Alaska Native	1 (4)		1(5)	
Asian	0 (0)		0 (0)	
Black	8 (31)		9 (43)	
Other[a]	2 (8)		1 (5)	
White	15 (58)		7 (33)	
Ethnicity		0.457		1
Hispanic or Latino	2 (8)		0 (0)	
Education		0.468		0.691
High school or less	9 (35)		4 (19)	
Some college	6 (23)		4 (19)	
Associate degree	4 (15)		0 (0)	
Bachelor's degree	5 (19)		6 (29)	
Master's or higher degree	2 (8)		3 (14)	
Baseline opioid use	2 (8)	0.460	1 (5)	0.877

[a]Participant self-identified as more than one race or declined to answer
[b]Compared to participants in cohort who completed follow-up

© 2022 Rodriguez IV et al. *JAMA Network Open.*
